# Intervention Effects of Group Sandplay Therapy on Children at Risk of Smartphone Addiction: Focusing on Internalizing and Externalizing Problems in the Korean Youth Self Report

**DOI:** 10.3390/children12050593

**Published:** 2025-05-01

**Authors:** Yang Hee Lee, Heajin Shin, Eunju Bae, Youngil Lee, Chang Min Lee, Se Hoon Shim, Min Sun Kim, Myung Ho Lim

**Affiliations:** 1 Department of Psychology, Graduate School, Dankook University, 119 Dandae-ro, Dongnam-gu, Cheonan 31116, Republic of Korea; parang88772@gmail.com (Y.H.L.); shins0422@hanmail.net (H.S.);; 2 Department of Anatomy, College of Medicine, Dankook University, 119 Dandae-ro, Dongnam-gu, Cheonan 31116, Republic of Korea; anat104@dku.edu; 3 Department of Neurology, College of Medicine, Dankook University, 119 Dandae-ro, Dongnam-gu, Cheonan 31116, Republic of Korea; nrdoc@dku.edu; 4 Department of Psychiatry, College of Medicine, Soonchunhyang University, Asan 31538, Republic of Korea; shshim2k@hanmail.net; 5 Department of Psychology and Psychotherapy, College of Health Science, Dankook University, 119 Dandae-ro, Dongnam-gu, Cheonan 31116, Republic of Korea

**Keywords:** smartphone addiction, group sandplay therapy, sandplay therapy, depression, somatic symptoms

## Abstract

**Objectives**: this study examined the intervention effects of group sandplay therapy (GST) on children at risk of smartphone addiction. **Methods**: The participants consisted of 113 elementary school students in grades 5 and 6 (ages 11–12), with 57 in the intervention group and 56 in the control group. The intervention group participated in the GST program once a week for 40 min over 10 weeks, while the control group received no intervention. The Youth Smartphone Addiction Scale (S-scale) and the Korean Youth Self Report (K-YSR) were used to assess the program. Additionally, a repeated measures ANOVA was employed to examine changes between pre- and post-tests. **Results**: compared to the control group, the intervention group that received GST showed a significant reduction in smartphone addiction (*F* = 7.355, *p* = 0.020), withdrawal/depression (*F* = 5.540, *p* = 0.032), and somatic symptoms (*F* = 4.542, *p* = 0.040) compared to the control group. **Conclusions**: GST was found to be effective in reducing smartphone addiction, depression, and somatic symptoms in children at risk for smartphone addiction.

## 1. Introduction

The rate of Internet access among Korean households is 99.9%, with a smartphone ownership rate of 96.1% among individuals aged six and older, indicating widespread usage across all age groups [1]. Smartphones have become an integral part of people’s lives, serving not only as tools for communication but also for accessing information and entertainment. However, high usage also increases the risk of smartphone overdependence. According to the 2023 Smartphone Overdependence Survey, 23.1% of the South Korean population is at risk of overdependence, up nearly 10% from 14.2% in 2014. The rate of overdependence among adolescents (ages 10–19) is 40.1%, which is significantly higher than that of children (25.0%) and adults (22.7%) [1].

Smartphone addiction has been defined as the overuse of smartphones to the extent that it disturbs the everyday lives of users [2]. Similar to other addictions, smartphone addiction can lead to increased tolerance, withdrawal symptoms, cravings, and loss of self-control [3,4,5].

Smartphone addiction can negatively impact both mental and physical health, causing various problems such as sleep disturbances, headaches, and dietary issues [6]. A meta-analysis by Sohn et al. [5] revealed that 23.3% of children and adolescents exhibited problematic smartphone use, leading to increased stress, poorer sleep quality, and higher levels of depression and anxiety. Ye et al. [7] found that Internet addiction exacerbates depression, which in turn can also worsen Internet addiction. Smartphone use has also been shown to be positively related to anxiety, with the two factors interacting over time to further intensify anxiety symptoms [8]. Among adolescents, higher smartphone dependence and fewer close friendships were associated with higher anxiety [9], leading to an increased risk of smartphone addiction [10]. Smartphone addiction has also been linked to aggressive behavior, anger, and hostility [11] and is associated with somatization and obsessive–compulsive symptoms [12]. Smartphone addiction has also been found to be associated with ADHD. Individuals with ADHD often exhibit impulsivity, low self-control, and heightened sensitivity to rewards. The characteristics of smartphones, such as instant feedback and portability, may cause adolescents with ADHD symptoms to become more dependent on their devices, thereby aggravating their symptoms [13,14]. Similar to Internet addiction, smartphone addiction has been linked to depression and anxiety [3,15], as well as other negative emotions such as loneliness. Furthermore, several studies have linked smartphone addiction to obsessive–compulsive disorder and ADHD [6,16,17]. Though similar to Internet addiction, smartphone addiction may pose a greater risk due to its accessibility at any time and place [18].

Smartphone addiction has been found to be associated with various psychological problems, including depression, anxiety, and aggression. In addition, adolescent characteristics, such as psychological immaturity and impulsivity, contribute to the risk of smartphone addiction [19]. Relationships with significant others also play a critical role. Negative relationships with parents, teachers, or peers are associated with a higher risk of smartphone addiction [20]. Conversely, positive and supportive relationships can help alleviate symptoms of depression and anxiety [21,22]. Thus, addressing smartphone addiction requires an integrated approach that considers psychological issues as well as social and environmental influences.

Non-pharmacological treatments for Internet addiction, such as cognitive behavioral therapy, educational interventions, combined interventions, and sandplay therapy, have demonstrated effectiveness in certain target groups. However, further research is needed to understand the impact and mechanisms of these interventions [23,24]. Sandplay therapy involves using sand and figures to express thoughts and feelings in a nondirective and receptive environment [25]. It can be particularly useful for children and patients who have difficulty expressing themselves verbally, as it allows them to visualize internal images using various figures, including people, animals, houses, and natural objects [26]. By engaging in play, sandplay therapy reduces psychological resistance and defense mechanisms [27] while relaxing the autonomic nervous system, which promotes interpersonal development and improves emotional regulation [28].

GST can enhance self-acceptance by enabling individuals to safely express their feelings and desires while experiencing acceptance and empathy from peers as they play in the sand [29]. Through interactions with group members, children can develop intimacy, acquire social skills, and achieve personal growth [30]. A meta-analysis by Roesler [26] found that sandplay therapy is effective in improving relationships, reducing social anxiety, and addressing ADHD, depression, anxiety, and addictive behaviors in children and adolescents. However, there is a lack of research on GST specifically for children with smartphone addiction. Therefore, this study investigated whether GST is effective in addressing smartphone addiction and internalizing/externalizing problems in affected children.

## 2. Methods

### 2.1. Study Design

This study was designed as a non-randomized controlled trial to assess the effectiveness of GST for children with smartphone addiction. The intervention group received GST, while the control group received no treatment. Both groups underwent pre- and post-tests. This study took place in a sandplay therapy room at an elementary school in Cheonan, South Chungcheong Province, South Korea. It was conducted over four semesters, from April 2021 to October 2022. Prior to the study, cooperation from elementary school teachers was requested, and three experts were consulted to develop a group plan. The program was conducted by a qualified expert with over ten years of experience in sandplay therapy under the clinical supervision of a pediatric psychiatrist.

### 2.2. Participants

A survey on smartphone addiction was conducted among 413 fifth and sixth graders at an elementary school in Cheonan, South Chungcheong Province, Republic of Korea. According to the survey results, 119 of the 413 students fell into the high-risk and potential-risk categories. Of these, five children refused to participate in sandplay therapy, and one child was excluded for other reasons. Parental consent was obtained from 113 students willing to participate in this study via school newsletters and individual contacts. Of the 113 final participants, 57 were assigned to the intervention group, while 56 were placed in the control group.

### 2.3. Intervention

The GST program consisted of ten 40 min sessions, held once a week on Tuesdays and Thursdays during after-school activities. The program was conducted in groups of 3 to 4 peers. Each child was provided with a sand tray measuring 72 cm wide, 57 cm high, and 7 cm deep. The figures for the GST program were shared among the children, and after completing their work, they took turns presenting and appreciating each other’s creations. The therapist facilitated the group interactions and encouraged the children to freely express their emotions. Throughout this process, the group members experienced mutual support, encouragement, and empathy (Table 1). The program was based on Boik and Goodwin’s [27] *Sandplay Therapy* and Kalff’s [31] Introduction to Sandplay Therapy, with reference to Kwak et al.’s [32] GST program for schools.

### 2.4. Measures

#### 2.4.1. Youth Smartphone Addiction Scale (S-Scale)

The S-scale is a standardized self-report scale developed by the National Information Society Agency [33] to measure smartphone addiction among adolescents aged 10 to 19. It consists of 15 items divided into four sub-factors. Factor 1 consists of 5 items related to impairment in daily life, Factor 2 consists of 2 items related to virtual world orientation, Factor 3 consists of 4 items on withdrawal, and Factor 4 consists of 4 items on tolerance. Scores range from 15 to 60 points. Elementary school students are considered high-risk if they meet one of the three following criteria: a total score of 42 or higher, a score of 14 or higher on Factor 1, or a score of 13 or higher on Factor 3. For potentially high-risk students, one of the four following criteria must be met: a total score between 39 and 41, a score of 13 or higher on Factor 1, a score of 12 or higher on Factor 3, or a score of 12 or higher on Factor 4. Each item is rated on a 4-point Likert scale, with responses ranging from “strongly disagree (1)” to “strongly agree (4).” To avoid response bias, items 8, 10, and 13 were reverse scored, with a higher total score indicating a greater likelihood of smartphone addiction. In this study, Cronbach’s α was 0.91.

#### 2.4.2. Korean Youth Self Report: K-YSR

The Youth Self Report (YSR) is a 119-item self-report test developed by Achenbach [34] for children and adolescents aged 11 to 18 years. Participants are asked to rate their emotional behavior on a 3-point Likert scale based on the preceding six months. The Korean Youth Self Report (K-YSR) was adapted and standardized by Oh et al. [35] and includes subscales measuring problem behavior syndrome and social competence syndrome. This study measured eight subscales, excluding other issues from the problem behavior syndrome. The eight subscales encompass internalizing problems such as anxiety, withdrawal, depression, and somatic symptoms. In addition, the subscales include externalizing problems such as aggressive and delinquent behaviors, as well as social problems and thought problems. The Cronbach’s α for the eight subscales in this study were as follows: anxiety/depression (0.87), withdrawal/depression (0.88), somatic symptoms (0.89), social problems (0.88), thought problems (0.87), attention problems (0.89), delinquent behavior (0.90), and aggressive behavior (0.88).

### 2.5. Statistical Analysis

Statistical analysis was performed using IBM SPSS Statistics 28.0. Additionally, an χ^2^ test and t-test were conducted for gender and age comparisons, respectively. Repeated measures ANOVA (RM-ANOVA) was used to examine changes between pre- and post-tests across groups after GST implementation.

### 2.6. Ethics Statement

This study was conducted in accordance with the Declaration of Helsinki and was approved by the Institutional Review Board of Dankook University (DKU 2020-03-004). Voluntary informed consent was obtained from all participants and their guardians after explaining the purpose and procedures of this study. Participants were also informed that they could withdraw from this study at any time without any penalty.

## 3. Results

### 3.1. Participant Demographics

**Figure 1:**
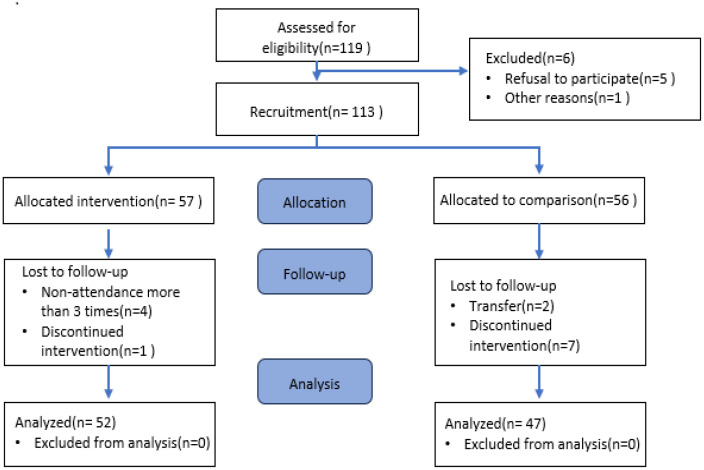
Flow diagram of participants.

Of the 113 children who participated in this study, 52 from the intervention group and 47 from the control group completed the post-test. Four children from the intervention group were excluded from the analysis: three who missed three or more sessions and one who did not complete the post-test. In the control group, nine children were excluded: seven who did not complete the post-test and two who transferred schools (see Figure 1).

Participants in the program consisted of fifth- and sixth-grade elementary school students (ages 11–12), with a mean age of 11.46 years (SD = 0.50) in the intervention group and 11.30 years (SD = 0.46) in the control group. Homogeneity tests indicated that the difference between the groups was not statistically significant (t = 8.83, *p* = 0.09). Additionally, the difference between genders was also not found to be significant (χ^2^ = 0.658, *p* = 0.42) (see Table 2).

### 3.2. Changes in K-YSR After GST Intervention

The analysis revealed significant effects for smartphone addiction related to group (*F* = 16.425, *p* < 0.001), time (*F* = 19.311, *p* < 0.001), and interaction (*F* = 7.355, *p* = 0.020). Withdrawal/depression was significant for the group (*F* = 5.540, *p* = 0.032) and time effects (*F* = 5.506, *p* = 0.021) but not for the interaction effect. Anxiety/depression and aggressive behavior were not significant for the group and interaction effects but were significant for the time effect (*F* = 10.685, *p* = 0.002). Somatic symptoms were not significant for group and time but were significant for the interaction effect (*F* = 4.542, *p* = 0.040). Thought problems were significant for the group effect (*F* = 4.359, *p* = 0.044) but not for the time and interaction effects. Delinquent behaviors, social problems, and attention problems were not found to be significant. Thus, the intervention group showed significant improvements in smartphone addiction, anxiety/depression, withdrawal/depression, somatic symptoms, aggressive behavior, and thought problems compared to the control group.

**Figure 2:**
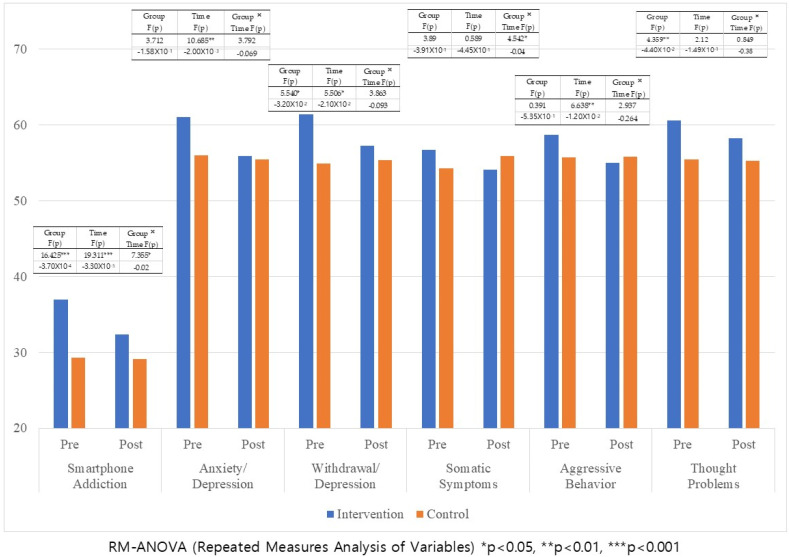
Differences between pre- and post-tests across intervention and control groups using RM-ANOVA (N = 99).

The differences between the intervention and control groups before and after the intervention were analyzed using repeated measures ANOVA (RM-ANOVA), and the results are presented in Table 3 and Figure 2.

## 4. Discussion and Conclusions

This study examined the effectiveness of GST intervention on children at risk of smartphone addiction. The results indicated significant improvements for the intervention group in smartphone addiction, anxiety/depression, withdrawal/depression, somatic symptoms, aggressive behavior, and thought problems.

First, the symptoms of smartphone addiction were found to be statistically significant in the group, time, and interaction effects following GST intervention. These findings are consistent with previous research on smartphone addiction among adolescents. Shin and Jang [36] reported significant decreases in smartphone addiction levels after a GST intervention with 78 middle school students. Additionally, Kim and Kim [29] found that GST improved peer relationships while reducing impulsivity and social anxiety in smartphone-addicted teenagers.

Smartphone addiction is associated with a lack of direct social support and difficulty in communicating and feeling connected with family and peers [37]. Limited social interaction has been shown to increase the likelihood of smartphone addiction, as it becomes more challenging to experience pleasure [38].

In addition, GST encourages children to express their feelings by communicating with one another about the various sensations of sand and the figures they place in the sand tray. Active peer interaction through GST may help alleviate symptoms of smartphone addiction by providing emotional support and fostering a sense of connection.

Furthermore, smartphone addiction has been linked to impairments in the brain’s inhibitory system, which regulates emotions and impulses [18]. Foo et al. [39] discovered that the prefrontal and temporal regions were co-activated during sandplay therapy, facilitating the recall and reprocessing of memories. In GST, sensory integration occurs as patients express the tactile sensations of touching the sand and the visual images of the figures they place in the sand tray. This process stimulates and activates the brain, strengthening neural connectivity.

Second, symptoms of anxiety and depression showed a significant effect over time after GST intervention, which was consistent with previous studies. A study by Lee et al. [40] on delinquent adolescents found that sandplay therapy significantly improved depression and anxiety symptoms in this population. Rousseau et al. [41] conducted sandplay therapy on 105 preschoolers aged 4 to 6 years living in a multiethnic neighborhood. The results showed that emotional issues such as depression and anxiety were alleviated.

GST integrates play into therapy, allowing children to express themselves freely using sand and figures without relying on verbal communication [42]. For children who have difficulty expressing themselves, this approach helps them process their memories and emotions more comfortably.

In the case of the withdrawal/depression variable, significant group and time effects were observed after GST, indicating a positive impact on the intervention group. Withdrawal and depression are often associated with peer relationships, as suggested by items such as “I feel lonely” and “I enjoy very few things”. GST helps alleviate depression by fostering peer attachment through interaction [29].

Third, GST showed a statistically significant interaction effect on somatic symptoms. Roubenzadeh and Abedin [43] used sandplay in a supportive environment with grieving adolescents, enabling them to understand, observe, and separate their emotions. This process contributed to a reduction in negative emotions such as depression, anger, and guilt, as well as somatic symptoms. Moreover, in a study of middle school students with PTSD symptoms, Cao et al. [44] reported that sandplay therapy effectively reduced somatic symptoms and negative emotions.

Fourth, the GST intervention resulted in significant changes in aggressive behavior. Lee et al. [45] reported that GST improved peer relationships and reduced behavioral problems such as aggression in a study of Korean–Chinese children. Abdollahi Keivani and Abolmaali [46] found that sandplay therapy could alleviate aggression, depression, and anxiety in preschool children. In addition, sandplay therapy has been shown to reduce aggressive behavior in female juvenile delinquents by providing an outlet for expressing their repressed anger and aggression [47]. This study suggests that aggressive behavior was mitigated by allowing these individuals to express negative emotions they were experiencing at the time by using sand and sharing their stories.

Fifth, GST resulted in significant between-group changes relating to thought problems. Shin and Park [48] found that GST significantly affected internalizing, externalizing, and cognitive issues among adolescents (grades 4–6) at a local children’s center. Von Gontard et al. [49] also reported that sandplay therapy significantly affected cognitive issues in a one-year study (with three assessment points of 0, 6, and 12 months) involving middle-class children and adolescents (ages 5–18). Thus, GST has been shown to be effective for addressing smartphone addiction, as well as for internalizing and externalizing symptoms.

However, this study has several limitations.

First, this study has methodological limitations in interpretation due to its non-randomized design and imbalanced gender distribution among participants.

Second, there were limitations in evaluating effectiveness due to relying solely on a self-report questionnaire. To establish more scientific evidence for the effectiveness of GST, additional studies involving brain wave observations and changes in autonomic nervous system activity are required.

Third, the current study was limited in its assessment of the long-term sustainability of the intervention. Future follow-up studies are warranted to evaluate the durability of treatment effects.

Fourth, this study’s scope was limited to a single elementary school, making it difficult to generalize the findings. Future research should expand to include different regions and age groups to explore the effects of GST on smartphone addiction comprehensively.

Fifth, this study was conducted over the course of four semesters, and although efforts were made to maintain consistent conditions, the extended intervention period posed limitations in fully controlling the environment. Therefore, future research should consider shortening the intervention period and utilizing regular class hours to ensure a more structured and stable setting for implementation.

Sixth, a limitation of the present study is its inability to control for factors such as parenting style and peer relationships, which are known to influence children’s emotional and behavioral development. For instance, the effectiveness of sandplay therapy may vary depending on the level of parental emotional responsiveness and the degree of peer support. Future research should incorporate assessments of the quality and frequency of parent–child interactions and peer relationships to more accurately examine the influence of these variables on therapeutic outcomes [20,50].

Lastly, the small sample size in this study limits the generalizability of the findings.

Nevertheless, this study is significant, as it validates the effectiveness of GST as an intervention for children with smartphone addiction within a context where research on this topic is scarce.

Group sandplay therapy (GST) facilitates emotional conflict resolution, emotional regulation, and the reconstruction and reorganization of the internal representational world by stabilizing the nervous system and promoting prefrontal cortex activation through nonverbal, sensory, and experiential approaches [30,51]. The findings of this study confirm the therapeutic mechanisms of GST to be effective in reducing smartphone addiction as well as various internalizing and externalizing symptoms.

This suggests that GST can be effectively applied not only in school settings but also within the home environment to improve interpersonal relationships and emotional regulation. Furthermore, in contemporary society where face-to-face interactions are decreasing due to the proliferation of online communication and artificial intelligence, such offline, physical, and sensory-based therapeutic approaches remain essential.

## Figures and Tables

**Table 1:** GST program highlights.

Directives	Session	Sandplay Therapy Activities
Departure	1	Feel the texture of the sand and express your thoughts and feelings.
Self-emotional contact	2	Close your eyes. Touch the sand and express the emotions that arise from its texture in the sand tray.
	3	Express the emotions you feel when recalling past memories.
Conflict and struggle	4	Express any negative emotions you’re experiencing right now.
	5	Recall and express an image of a hero overcoming hardship and adversity.
Family, friends, school	6	Express your feelings while thinking about your family.
	7	Express your thoughts and feelings about friends and school.
Self-understanding and acceptance	8	Express yourself in the sand tray.
	9	Recall the sandwork you’ve created thus far and express your feelings.
Integration	10	Imagine and express a new version of yourself.

**Table 2:** Demographics of the intervention group (n = 52) and control group (n = 47).

Variables	Intervention (n = 52)	Control (n = 47)	t/χ²	*p* Value
Age	11.46 ± 0.50	11.30 ± 0.46	8.83	0.09
Sex			0.658	0.42
*Male*	18 (34.6)	20 (42.6)		
*Female*	34 (65.4)	27 (57.4)		

**Table 3:** Differences between pre- and post-tests across intervention and control groups using RM-ANOVA (N = 99).

Variables	Group (N)	Mean ± SD					
		Pre	Post	Group F (*p*)	Time F (*p*)	Group × Time F (*p*)	η^2^ Interaction
Smartphone Addiction	Intervention (52)	36.98 ± 6.18	32.35 ± 6.38	16.425 *** (3.7 × 10^−4^)	19.311 *** (3.3 × 10^−5^)	7.355 * (0.020)	0.071
	Control (47)	29.30 ± 4.65	29.15 ± 6.72				
Anxiety/Depression	Intervention (52)	61.02 ± 10.08	55.92 ± 9.99	3.712 (0.158)	10.685 ** (0.002)	3.792 (0.069)	0.038
	Control (47)	55.98 ± 9.47	55.45 ± 8.77				
Withdrawal/Depression	Intervention (52)	61.35 ± 10.60	57.21 ± 9.86	5.540 * (0.032)	5.506 * (0.021)	3.863 (0.093)	0.038
	Control (47)	54.85 ± 8.28	55.36 ± 7.88				
Somatic Symptoms	Intervention (52)	56.73 ± 6.52	54.10 ± 7.23	3.89 (0.391)	0.589 (0.445)	4.542 * (0.040)	0.045
	Control (47)	54.28 ± 7.05	55.89 ± 7.77				
Delinquent Behavior	Intervention (52)	55.46 ± 7.20	54.90 ± 6.53	2.149 (0.278)	0.080 (0.778)	2.332 (0.453)	0.023
	Control (47)	53.43 ± 4.17	54.38 ± 5.46				
Aggressive Behavior	Intervention (52)	58.71 ± 8.40	54.98 ± 7.44	0.391 (0.535)	6.638 ** (0.012)	2.937 (0.264)	0.029
	Control (47)	55.74 ± 7.07	55.83 ± 7.79				
Social Problems	Intervention (52)	60.15 ± 8.36	58.19 ± 9.76	1.069 (0.333)	1.771 (0.187)	4.865 (0.314)	0.048
	Control (47)	55.62 ± 7.99	57.21 ± 8.90				
Thought Problems	Intervention (52)	60.54 ± 9.25	58.19 ± 9.76	4.359 ** (0.044)	2.120 (0.149)	0.849 (0.380)	0.036
	Control (47)	55.47 ± 7.35	55.28 ± 8.43				
Attention Problems	Intervention (52)	57.46 ± 9.57	53.77 ± 7.53	2.900 (0.134)	3.485 (0.066)	2.447 (0.232)	0.025
	Control (47)	52.64 ± 5.36	53.04 ± 6.47				

RM-ANOVA (repeated measures analysis of variance); * p < 0.05, ** p < 0.01, *** *p* < 0.001.

## Data Availability

The datasets generated during and/or analyzed during the current study are available from the corresponding author on reasonable request.
